# Exploring experiences of loneliness among Canadian long‐term care residents during the COVID‐19 pandemic: A qualitative study

**DOI:** 10.1111/opn.12509

**Published:** 2022-11-08

**Authors:** Chelsea B. Smith, Karen Lok Yi Wong, Flora To‐Miles, Sheila Dunn, Mario Gregorio, Lily Wong, Samantha Tam, Polly Huynh, Lillian Hung

**Affiliations:** ^1^ IDEA Lab University of British Columbia Vancouver British Columbia Canada; ^2^ Community Engagement Advisory Network Vancouver British Columbia Canada; ^3^ Richmond Home and Community Vancouver Coastal Health Vancouver British Columbia Canada

**Keywords:** COVID‐19, loneliness, long‐term care, qualitative research, resident experience, social isolation

## Abstract

**Background:**

The COVID‐19 pandemic has significant impact on long‐term care (LTC) residents’ health and well‐being.

**Objectives:**

This study investigated resident experiences of loneliness during the COVID‐19 pandemic in Canadian LTC homes to offer lessons learned and implications.

**Methods:**

15 residents and 16 staff members were recruited from two large urban Canadian LTC homes with large outbreaks and fatalities. We used a telepresence robot to conduct one‐on‐one semi‐structured interviews with participants remotely. We applied the Collaborative Action Research (CAR) methodology and report the early phase of CAR focused on collecting data and reporting findings to inform actions for change. Thematic analysis was performed to identify themes.

**Results:**

Four themes were identified. The first two themes characterise what commonly generated feelings of loneliness amongst residents, including (1) social isolation and missing their family and friends and (2) feeling hopeless and grieving for lives lost. The second two themes describe what helped residents alleviate loneliness, including (3) social support and (4) creating opportunities for recreation and promoting positivity.

**Conclusions:**

Residents living in LTC experienced significant social isolation and grief during the pandemic that resulted in loneliness and other negative health consequences.

**Implications for practice:**

Promoting meaningful connection, safe recreational activities and a positive atmosphere in LTC homes during the pandemic may help mitigate residents’ experiences of loneliness due to social isolation and/or grief and enhance their quality of life.

**Summary statement of implications for practice**

**What does this research add to existing knowledge in gerontology?**

- This is the first paper to our knowledge that contributes perspectives on resident loneliness during the COVID‐19 pandemic from both long‐term care residents and a diverse range of staff members via one‐on‐one interviews.
- Summarises effective strategies in reducing resident loneliness in long‐term care during the COVID‐19 pandemic.
- Worked with an older adult living with dementia and family partners to provide the first paper to our knowledge focused on a Canadian perspective and context for this particular topic and scope.

**What are the implications of this new knowledge for nursing care with older people?**

- Describes how understaffing, safety protocols, COVID‐19 spread and visitation limitations contributed to residents' experiences of social isolation and grief.
- Highlights the impact of social isolation, missing family and friends, and grief on resident loneliness to better understand residents' experiences of loneliness in LTC throughout the COVID‐19 pandemic
- Emphasises the importance of offering different forms of social support, creating opportunities for recreation and promoting a positive atmosphere in LTC settings to help mitigate resident loneliness.

**How could the findings be used to influence policy or practice or research or education?**

- Influences policy and practice by providing a summary of resident and staff experiences to inform decisions related to new infection control protocols, support programs and educational programs for staff.
- Encourages stakeholders in LTC to implement new incentives that promote social support, modified recreation and a positive atmosphere to mitigate resident loneliness.
- Provides a foundation for future research focusing on investigating and testing different tools to mitigate resident loneliness in LTC.

## 1 INTRODUCTION

Loneliness is a subjective experience in which a person feels a lack of meaningful connections and relationships (McMullan et al., 2020), and the loneliness of older adults is associated with many negative health conditions. For example, loneliness is associated with hearing loss (Sung et al., 2016), pain (Emerson et al., 2018), poorer perceived health (Coyle & Dugan, 2012), earlier deaths (Luo & Waite, 2014), an increase in mental health problems (Coyle & Dugan, 2012), depression (Gonyea et al., 2018), the use of opioids and benzodiazepines (Vyas et al., 2021), worsening cognition (Donovan et al., 2017) and poorer sleep (Shankar, 2020).

Older adults who are socially isolated are more likely to feel lonely (McMullan et al., 2020; Park et al., 2019; Rasnaca et al., 2022; Toepoel, 2013). Despite loneliness and social isolation being interrelated, they are different concepts. While loneliness is a subjective experience, social isolation is an objective situation in which a person has a small social network, reduced contact with other people and limited participation in social activities (Silva et al., 2022).

Prior to the COVID‐19 pandemic, older adults were already more socially isolated in long‐term care (LTC) settings than community settings, leading to increased loneliness. Prieto‐Flores et al. (2011) conducted a survey study suggesting that older adults living in care settings are more likely to feel lonely than those who live in the community due to fewer opportunities to connect with family, friends and neighbours than those living in the community. Huang et al. (2022) used interviews to understand older residents' perceptions of loneliness in LTC facilities in Taiwan. They found four themes: ‘being cut off from continually meaningful relationships’, ‘experiencing tears of pain’, ‘feeling alone’ and ‘lacking a sense of belonging’. Jansson et al. (2022) used ethnography to explore the experience of loneliness of residents in LTC in Finland. They looked into three types of loneliness: social, emotional and existential loneliness. Although Huang et al. and Jansson et al. are recent studies, they are not focused on loneliness during the COVID‐19 pandemic.

Since the beginning of the COVID‐19 pandemic, many LTC homes across the world have followed public health measures to mitigate the spread of the virus and protect residents and staff. Efforts to reduce the spread of COVID‐19 include limiting visitors from outside the care home, restricting recreational activities, and in some extreme cases, isolating residents in their rooms. Unfortunately, these health measures have increased levels of social isolation among LTC residents and therefore levels of loneliness. Rasnaca et al. (2022) suggest that loneliness is especially prevalent among older adults in LTC in Latvia, a country in north‐eastern Europe, since the COVID‐19 pandemic due to visitation restrictions. Simard and Volicer (2020) also suggest that residents in LTC homes in Australia have been particularly lonely during COVID‐19 due to visitation restrictions and paused group activities. Van der Roest et al. (2020) examined levels of loneliness during visitation restrictions related to COVID‐19 in LTC facilities in the Netherland and found that there was a high percentage (77%) of residents who felt lonely. Aho (2022) from the United States suggests the COVID‐19 lockdown in LTC intensified the lonely feeling of residents. Beogo et al. (2022) conducted a scoping review argue that loneliness existed among residents in LTC in Canada before COVID‐19 and the global pandemic further exacerbated the problem because residents were more isolated.

Loneliness is an important topic in LTC settings and has been exacerbated by the COVID‐19 pandemic. There is limited research that allow residents to voice their experiences even though they are directly affected. It is important to understand experiences of loneliness from the residents' perspectives themselves to properly inform implementations and policy. This paper will explore residents' experiences of loneliness during the COVID‐19 pandemic by using a telepresence robot. The use of a telepresence robot allowed the study team to interview residents remotely during a time of visitation restriction. Because staff work closely with residents and have witnessed their experiences throughout the pandemic, the perspectives of staff on residents' loneliness is included in the study to gain richness and depth in interpretation. We took a Collaborative Action Research (CAR) approach, which is underpinned by concepts of social construction in meaning, multiple truths and knowledge co‐production for change. Gathering insights from both staff and residents allowed us to explore multiple perspectives on the complex experiences of loneliness among residents. This is in line with ‘crystallisation’ (Ellingson, 2014)—an approach used by modern qualitative scholars; polyvocality adds power, richness, and depth of understanding in CAR, which includes people with differing perspectives on the issues being addressed.

## 2 METHODS

### 2.1 Study design

We applied the Collaborative Action Research (CAR) methodology (Traynor et al., 2006) which typically has cycles of planning, acting, observing and reflecting. The first phase involves recruitment and approval of the study. The results of early cycles help to reveal relevant issues and priorities to inform subsequent actions. CAR is useful and appropriate to engage multiple stakeholders to understand and co‐design interventions for complex issues. This paper reports data collection and analysis of the early findings about residents' experiences, priority needs and possible strategies. Our goal was to (1) understand the experiences of loneliness for residents during the COVID‐19 pandemic and (2) report what interventions were considered effective in mitigating resident loneliness. These findings will be used to inform the next phase of CAR focused on implementation, specifically co‐developing and implementing actions and policy with stakeholders.

### 2.2 Research setting and participants

The study took place in two large urban Canadian LTC homes with large COVID‐19 outbreaks and fatalities (Mackenzie, 2021). A large outbreak in a LTC home refers to more than 26 cases of residents who caught COVID‐19 (Mackenzie, 2021). One institution had a major outbreak in the summer of 2020, and the other institution had a major outbreak between December 2021 and February 2022. There were multiple smaller outbreaks at both of the LTC homes, and our study includes experiences from both outbreak and non‐outbreak situations during the COVID‐19 pandemic. We chose these two LTC homes because they both had large outbreaks and we wanted to examine extreme cases (i.e. LTC homes which were severely impacted by COVID‐19). The resident population of these LTC homes is multicultural and has various complex needs, requiring 24‐h nursing care.

We interviewed both older adults living in the LTC homes and staff members (all staff including frontline workers, administration and managers were invited to participate) working in the LTC homes. The inclusion criteria were English‐speaking residents and staff in the care homes who were able to understand the research purpose and procedures. There was no specific exclusion criterion. A convenient sampling method was used for recruitment; study posters were used to invite participants, and the recreation staff members also helped recruit participants.

We gave the inclusion criteria to staff. The staff decided which residents would meet the inclusion criteria according to their professional knowledge and relational understanding of residents. They introduced the study and explained its purpose to these residents in simple language. They assessed if residents could understand with reference to their professional judgement. If residents could understand and agreed to participate, they would connect them to the research team. Before starting the interview, the interviewer would use a script to introduce the study and explain its purpose to the residents again to ensure residents understood and were interested in participating. We did not screen participants for cognitive impairments or dementia. Due to COVID‐19, we were not able to go to the facilities and conduct cognitive tests. We did not ask staff to conduct cognitive tests because we did not want to add workload during a time of staff shortage. We also wanted to be inclusive and did not want to exclude residents based on dementia or cognitive impairment.

Sufficient data or ‘information power’ (Varpio et al., 2017) was obtained to answer our research questions after 15 residents and 16 staff members (nurses, care workers, music therapists, recreation staff, administrators and directors) were interviewed. Two staff members dropped out of the study due to busy schedules. The perspectives of both residents and staff members were included to gain more insights to enhance research, thereby developing a more comprehensive understanding based on plurality and multiple viewpoints (Chamberlain et al., 2011; Tracy, 2010).

### 2.3 Research team and instruments

The members of our research team included seven women and one man (MG). We strived to conduct a patient‐oriented research study, and thus, our research team included one community‐dwelling older adult living with mild dementia (MG) and two family partners (i.e. have family members living in LTC; SD and LW). Patient‐oriented research focuses on engaging patients, their caregivers and families as partners in the research process (Frisch et al., 2020). Although we do not specifically target individuals living with dementia or family members of LTC residents in our interviews, these members of the research team bring important lived experiences and perspectives that the other researchers do not have. They were equipped with the skills to work within our research team and provide important alternative perspectives compared to the other traditionally academically trained researchers on the team. There are residents in LTC homes that are living with dementia and other cognitive impairments, and we wanted to include this perspective during analysis to promote the rigour of the research. The research team members with a family member in LTC provided important insight as they understood the difficulties LTC homes experienced during the pandemic. They helped the entire research team better understand the experiences of residents and staff. As researchers, we wanted to include people in the conversation who more closely relate to and understand the population we study.

Interviews were conducted by CS (BSc, Research Assistant), KW (MA, MSW, Research Assistant), FT (PhD, Research Assistant), SD (family partner), LW (family partner) and LH (RN, PhD, Assistant Professor). The family partners completed seven of the 15 resident interviews. The staff interviews and remainder of resident interviews were completed by the research assistants. All interviewers had training and/or prior experience in qualitative research methods. No relationship was established with participants prior to the study. Participants understood their interviewer was part of the research team investigating loneliness in LTC.

### 2.4 Data collection procedure

We conducted individual interviews by videoconferencing through a telepresence robot (Double Robotics, 2022) or calling over the phone, due to limitations on visitation in the care homes. All of the residents completed the interviews through a telepresence robot since some did not have access to a phone; an advantage of using telepresence robots was that residents were able to have a visual of the interviewer, which facilitated the interviews more successfully. Staff assisted in setting up the robots for residents to use for the interviews. The staff members completed the interview over the phone, which was more convenient (over telepresence robots that required setting up) due to busy shifts.

Staff and residents were in the LTC home during the interview. Staff ensured that residents could interview in their room or a different private room in the facility. Phone interviews were organised for the staff when they had time and a quiet place to complete the call; managers were able to complete the phone interviews in their private offices.

The questions asked during interviews are provided in Appendix A. The interviews were conducted in September–December 2021, each lasting for approximately 30–60 min. These interviews occurred after the major and smaller outbreaks each LTC setting experienced. Although the interviews were conducted during a non‐outbreak setting, participants were encouraged to share experiences from both outbreak and non‐outbreak settings over the course of the COVID‐19 pandemic. Data were audio recorded and transcribed verbatim. Field notes were made during and after interviews.

### 2.5 Data analysis

**FIGURE 1:**
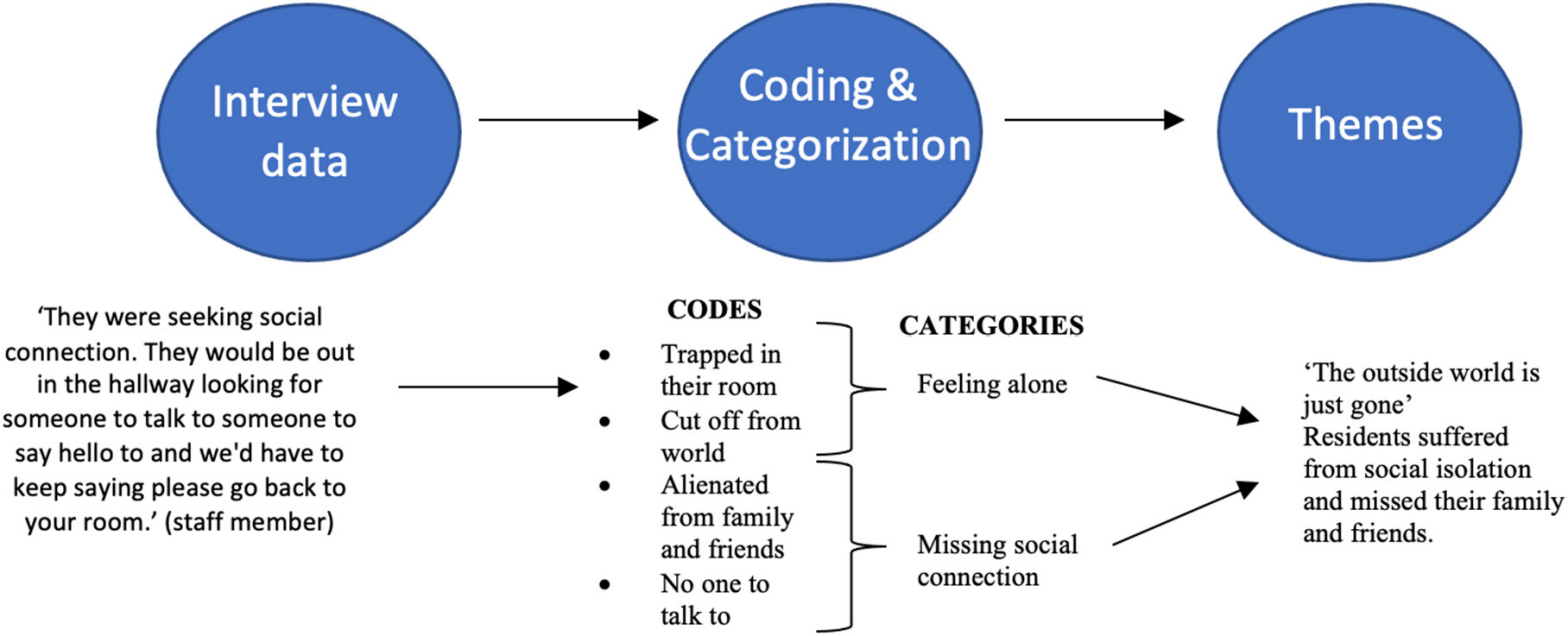
Process of theme development. Original quotations from interview data were given descriptive codes. After all of the data was coded, related codes were grouped together into a general category. Two or more categories are grouped together to create the overarching theme.

We performed thematic analysis to identify themes using an inductive coding approach (Braun & Clarke, 2008). The analysis was completed in five steps: (1) interview transcriptions were read independently; (2) interview transcriptions were discussed in research team meetings so research team members with different backgrounds (i.e. clinical researcher, family partners, older adult with lived experience of dementia, social worker and research assistant) could offer their interpretation of the data; (3) a researcher (CS) developed initial inductive codes and collated the data relevant to each code; (4) a researcher (CS) grouped the codes to generate categories; (5) a researcher (CS) grouped related categories to form descriptive themes and incorporated a direct quotation into the theme name that is reflective of the data (see Table 1 and Figure 1); (6) the whole team discussed the themes together in research meetings and gained analytic consensus.

**TABLE 1:** Theme development

Themes	Categories	Codes	Examples of original quotations within this theme
‘The outside world is just gone’ Residents suffered from social isolation and missed their family and friends	1. Feeling alone 2. Lacking meaningful relationships 3. Missing social connection	Trapped in their room No one to talk to Always on their own Cut‐off from world No meaningful friendships Alienated from family and friends Missing loved ones Wanting to leave LTC home	‘What I found was once you come here the outside world is just gone. I've been so isolated here it's unbelievable’. (resident) ‘They were seeking social connection. They would be out in the hallway looking for someone to talk to someone to say hello to and we'd have to keep saying please go back to your room’. (staff member)
‘Straight down the hallway they all passed away … it's like a nightmare’ Residents felt hopeless and grieved for lives lost	1. Grief 2. Feeling hopeless	Shocked by deaths Mourning Loss of friends and peers Feeling upset Losing hope Lack of control over situation	‘Straight down the hallway they all passed away … it was a wave of shock … it's like a nightmare’. (resident) ‘Residents had an opportunity to share their grief, because some of the people who had passed away with their friends or roommate or the person that they used to sit with at the table’. (staff member)
‘Number one is people you know, connection with people’ Social support reduced resident loneliness	1. Meaningful social connection 2. Acknowledgment 3. Safe social support	Virtual connection Companionship with other residents Empathy Relationship with staff members Validation Acts of kindness	‘Number one is people you know, connection with people’. (resident) ‘just holding their hand makes a difference … telling them they haven't been forgotten by their family’. (staff)
‘I have to live positive’ Creating opportunities for recreation and promoting positivity reduced loneliness	1. Positive atmosphere 2. Self‐fulfilment 3. Recreation	Engagement Enthusiasm Solo hobbies Keep themselves busy Pleasant physical atmosphere Staff meeting resident's needs Positivity Modifying recreation	‘I want to learn so I can keep up with the world … I have to live positive’. (resident) ‘they will put on these different game shows … the residents they came alive, they loved it’. (staff member)

*Note*: Each theme encompasses two or more categories. Examples of codes that were grouped to form categories within each theme are listed.

### 2.6 Ethics considerations

The study was approved (H21‐01438) by the Research Ethics Board at the University of British Columbia and the local health authority. All participants gave informed consent in both oral and written form prior to the interview, and we followed an ongoing consent and assent approach (Dewing, 2007; Hung et al., 2017); each time researchers start a research activity with participants, we would reiterate the study procedures and watch for any behaviours or indications that show that they do not want to participate, and we ensure that they know they have the choice to withdraw from the study. We have used pseudonyms throughout the reporting of data to maintain confidentiality.

## 3 RESULTS

We interviewed 15 residents and 16 staff members (nurses, care workers, music therapists, recreation staff, administrators and directors). Table 2 summarises the descriptive characteristics of the participants.

**TABLE 2:** Descriptive characteristics of participants

Resident characteristics %	%	Staff characteristics	%
Age (years)		Age (years)	
60–75	10	Younger than 35	20
76–85	80	36–50	60
Older than 85	10	Older than 50	20
Gender		Gender	
Male	40	Male	20
Female	60	Female	80
Ethnicity		Ethnicity	
Caucasian	80	Caucasian	40
South Asian	20	South Asian	60

**FIGURE 2:**
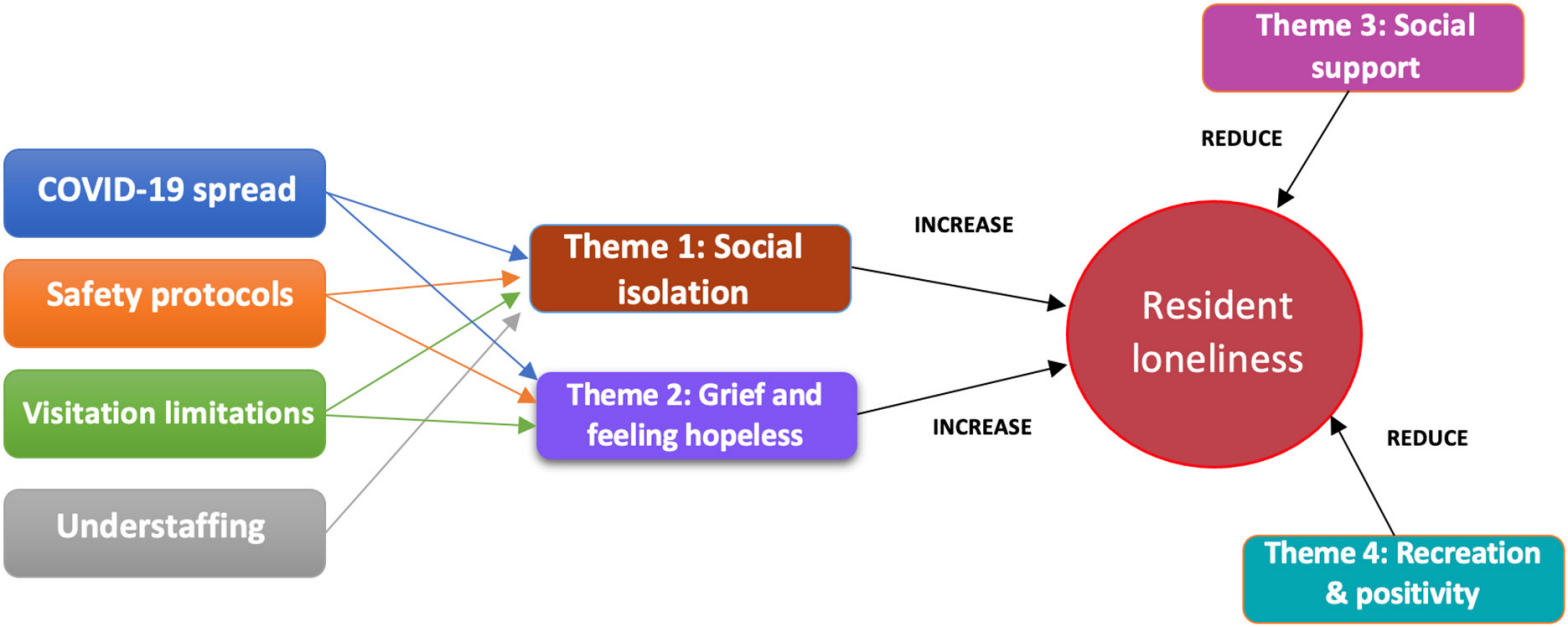
Summary of the four identified themes and the four factors related to the pandemic that contributed to Themes 1 and 2.

Thematic analysis revealed four themes related to residents' experiences of loneliness during the pandemic. The findings align with our two goals within the phase two process of the CAR methodology: (1) understand the experience of loneliness for residents during the COVID‐19 pandemic and (2) report what interventions were considered effective in mitigating resident loneliness. The first two themes describe what commonly generated feelings of loneliness among residents, including (1) residents suffered from social isolation and missed their family and friends and (2) residents grieved for lives lost and feared the consequences of the COVID‐19 pandemic. The interviews pointed towards four main factors that contributed to social isolation and grief: COVID‐19 spread, safety protocols, visitation limitations and understaffing. The second two themes describe what helped residents alleviate loneliness, including (3) social support and (4) creating opportunities for recreation and promoting positivity. See Figure 2 for summary of themes.

### 3.1 Theme 1: ‘The outside world is just gone’: Residents suffered from social isolation and missed their family and friends

#### 3.1.1 Resident perspective

Residents were confined in the care homes for most of the pandemic and spent two and a half months isolated in their rooms during outbreaks. Visitation was restricted, and from the resident perspective, there was a strong sense of social isolation that resulted in loneliness. One particular resident, Walter, explained his experience as ‘very isolating … it's like jail’. Another resident, Mary, similarly described, ‘it was very lonely with the COVID‐19. You couldn't go into anyone's room, and we couldn't see our friends. And our family couldn't come and visit so it was very lonely’. Walter and Mary's description captured a sense of social isolation described by many residents. Mary also highlighted the strong sense of disconnection with family and friends many residents experienced because of visitation restrictions. This theme was present even in discussions about life during non‐outbreak settings, as residents described feeling disconnected from others with the ongoing limitations on visitation and mobility between areas in the care home.

Notably, there were some residents who felt socially isolated but did not describe their experience as lonely. These residents explained they were not lonely because they were always surrounded by staff. For example, Leo shared ‘there's always nurses around here so you don't have time to get lonely’; however, further described he had not seen his friends in over two years and was eager to move out of the care home and into the community to reunite with them. Another resident, Amanda, shared that she did not feel lonely herself but felt some residents may be hesitant to express how lonely they feel. She expressed, ‘some people do not like to share they feel the loneliness because they're afraid to accept and to speak it out’.

#### 3.1.2 Staff perspective

Residents were seen as socially isolated from the staff perspective. The extent and impact of social isolation on levels of loneliness seemed more extreme from the staff perspective. For example, one staff member, Sandra (manager), shared ‘many of the residents said I'd rather die of COVID than be here alone’. Multiple other staff members described hearing a similar perspective from residents who were more interested in reuniting with their friends and family than isolating in the LTC home by themselves.

Staff also felt some residents grew so lonely that they would try to leave their room despite understanding the threat of the virus. Isolation protocols were also ineffective for residents with dementia who constantly tried to leave their rooms to seek someone to talk to. Staff recognised further consequences of social isolation in residents' final moments before they died as they cried and called out to their family members.

For many residents who survived the major outbreaks, staff felt the impact of the isolation was persistent. Residents, however, did not report on this aspect. One staff member, Evelyn (care aide), felt residents ‘took a downturn … physical, mental, all aspects of their life’. Staff member Lucy (manager) shared ‘they would stop eating, almost give up’. From the staff perspective, the mental and physical health of residents noticeably deteriorated, and many did not recover. After the major outbreaks, staff recalled some residents did not want to leave their rooms because, as Evelyn described, they ‘didn't see the point’. Staff reported residents' walking became worse, and many required a wheelchair after they were isolated in their rooms for two and a half months. Staff noticed new psychiatric problems arise in some residents as well (e.g. hallucinations). See Appendix A for stories shared by staff member Rachel (nurse) about two residents who struggled with loneliness due to social isolation and unfortunately died. Although the cause of death of the two residents described in Appendix A cannot be confirmed, the stories illustrate how the staff member perceived the residents and their deaths. Overall, residents experienced social isolation and missed their family and friends with lasting effects that persisted even long after the outbreaks were over.

### 3.2 Theme 2: ‘Straight down the hallway they all passed away … it's like a nightmare’: Residents felt hopeless and grieved for lives lost

#### 3.2.1 Resident perspective

In both care homes, COVID‐19 caused a substantial number of deaths among residents. Residents lost friends, neighbours and/or tablemates. The lack of control residents had over their situation and the impact of COVID‐19 exacerbated feelings of hopelessness and loneliness. In addition to social isolation and missing friends and family, residents also processed grief for the overwhelming number of deaths. For example, resident Amanda reported, ‘all of a sudden, I find out quite a few of the seniors that I know … they all passed away … it was a wave of shock … cause they all say hey let's go out for dinner tomorrow … and then they all pass away’. She reported it was ‘like a nightmare’, demonstrating the feelings of despair residents experienced. Another resident, Mary, also discussed the consequences of losing friends in the care home, ‘some of the people I knew died during COVID‐19 and I miss them. It was lonely’. Furthermore, the husband of a resident named Roberta died, and as a result, she reported feeling extremely lonely and empty. She was ‘worried he left her forever’ and felt like her last meaningful relationship was gone. Losing individual residents normally saw every day contributed to feelings of hopelessness and loneliness.

#### 3.2.2 Staff perspective

From the staff perspective, residents did not only grieve for lives lost, but also grieved because they were not able to see their loved ones due to COVID‐19 safety protocols. As a result, staff emphasised that residents experienced feelings of sadness and despair. For example, while grieving for lost connection with family members, staff member Anastasia (nurse) shared that residents were ‘agitated’, ‘restless’ and ‘crying’. These are considered to be signs of grief.

In response to these events, the care homes organised Celebration of Life ceremonies to recognise the residents who passed. These were held virtually and provided residents an opportunity to mourn. Although the residents did not mention the ceremonies, the staff revealed that they seemed to help both residents and staff cope with grief. One care home also brought in a grief specialist who ran workshops for the different steps of processing grief, which staff member Devon (recreation staff) described was helpful to residents and staff because they could work through their emotions and come to terms with losing people they knew and cared about.

### 3.3 Theme 3: ‘Number one is people you know, connection with people’: Social support reduced resident loneliness

#### 3.3.1 Resident perspective

Socialisation even brief connection with staff or a call with family or friends was meaningful during outbreak conditions. Residents missed their loved ones outside of the LTC home because visitation was restricted throughout the pandemic. For some residents, there was no one outside of the care home they could contact. Instead, they found social connection by building relationships with staff and other residents. The importance of residents' relationships with staff was emphasised through the resident interviews. For example, when resident Mary was speaking about a care aide, she said, ‘she's my best friend … she is a wonderful lady. We all need friends, right?’ Nearly all of the other residents reported similar appreciation and connection to the staff. For some residents, they felt the staff were the only people they could trust and rely on. Mary also noted that not all staff members were socially connected to residents because they did not have time to socialise. When talking about the staff, she mentioned ‘most are good. Some are difficult, they are very busy. They have 100 people to look after. By the time they look after them all they don't have a lot of time for socializing’. Given the staff shortages and the large workload many LTC staff members carry, particularly during COVID‐19, some staff members may be overwhelmed and struggle to dedicate time to connect to residents.

When residents were no longer confined to their rooms, many benefited from socialising with other residents. This was not the case for all residents, however. For example, Walter (resident) has high cognitive functioning and shared he struggled to find other residents on his floor to converse with. Another resident, Carol, expressed that she found it difficult to make friends with the residents on her floor because they spoke a different language and were unable to communicate with her.

For residents who had someone to contact outside of the home, connection was facilitated by phone and video calls. Some residents owned tablets or phones and were able to make daily calls to loved ones, which helped reduce their loneliness. For example, Carol reported, ‘I don't think I've ever felt lonely. I just pick up the phone’. For others, staff members had approximately six tablets for the facility and used an online booking system to schedule virtual visits. The residents who used the technology found it effective in connecting them with their loved ones.

#### 3.3.2 Staff perspective

The staff emphasised the importance of connecting with residents as much as possible, although maintaining meaningful connections was difficult during the pandemic due to understaffing. Understaffing was a factor cited by all staff members that made connecting with residents more challenging. Yet, staff recognised how essential their role is in supporting residents. For example, staff member Rachel (nurse) expressed, ‘holding their hand and giving them some time, 5–10 min of time, will surely make a difference … just them having reassurance that you are able to come back and be available to them when they need it the most, I think it's enough for them to get through the day’. Rachel further highlighted the importance of staff in resident well‐being, ‘we have to get to know them at a personal level. Their likes and their dislikes, their past life, hobbies’. This mitigated resident loneliness from the staff perspective.

Staff emphasised how helpful video calls were to the residents and revealed virtual visits helped connect residents to family members who do not live near the care home. Furthermore, staff members mentioned that when visitation was allowed, designated outdoor spaces permitted visitors to socialise with residents from a distance. There were also window visits where visitors would have face‐to‐face connection with the resident. Various opportunities for connection were important for helping mitigate resident loneliness from the staff perspective.

### 3.4 Theme 4: ‘I have to live positive’: Creating opportunities for recreation and promoting positivity reduced loneliness

#### 3.4.1 Resident perspective

Creating a setting where residents were busy and engaged helped reduce loneliness from the resident perspective. Residents were excited by activities that gave them purpose and helped connect them to what they enjoy. Unfortunately, many recreational activities were paused during outbreaks and modified throughout the rest of the pandemic. Residents reported adapted recreational activities, such as those outlined in Table 3, reduced their loneliness. Providing small resources in each room during the outbreak, such as a TV, art kit, radio or collection of books, allowed residents to experience joy while being cut‐off from the rest of the home. In non‐outbreak situations, residents were able to feel less lonely as they were able to reengage in activities such as socially distanced happy hour, music therapy and outdoor concerts. Residents also reported positivity was an important factor in reducing loneliness and promoting engagement with their daily activities. One particular resident who showed pride in her independence, Amanda, explained ‘I have to live positive’. When residents felt optimistic about their situation, they felt less lonely.

**TABLE 3:** Adapted recreation activities for outbreak vs. non‐outbreak settings

Setting	Activities
Outbreak	Connecting with pastor or religious service virtually Staff singing or reading stories and poems at residents' doors Listening to stereo Watching TV Reading Creating art Word puzzles and colouring pages Staff performing skits and singing karaoke in hallway Matching residents to volunteers and facilitating virtual connections Christmas cards in different languages made by local schoolchildren
Non‐outbreak	Guests streaming in virtually rather than in‐person (music therapy, exercise classes, Happy Hour guest) Socially distanced activities in communal area within one neighbourhood (~13 residents) Music therapist singing at residents' doors or in hallway Scenic drives Outdoor concerts (residents watch from balcony) Matching residents to volunteers and facilitating virtual connections Going outside for a walk

#### 3.4.2 Staff perspective

From the staff perspective, staff members strived to find ways to create a positive atmosphere using recreation and encouragement. These activities helped alleviate resident loneliness by creating what one staff member, Chad (manager), described as ‘moments of joy’. Another staff member, Stephanie (care aide), emphasised the importance of creating a positive physical atmosphere as well. She suggested residents would benefit from planted flowers because they could touch and smell them, and watch them grow. Staff member Evelyn (care aide) further emphasised creating a ‘more cheery atmosphere’ by using brighter colours on the walls so residents ‘feel comfortable here. They should feel that it's warm and welcoming than just a hospital’. Stephanie also emphasised the importance of inclusion when creating a positive environment, such as learning and teaching songs from the cultures of different residents. She highlighted this is important to ensure no one feels forgotten, which contributes to loneliness.

In one of the LTC facilities, a positive atmosphere was further promoted when staff worked in small and consistent groups called ‘cohorts’. The cohorts allowed staff members to work in the same part of the LTC home with the same group of people over time. During a period with reduced socialisation across the LTC home, it allowed staff to get to know each other and the residents better, rather than working with new people each day. The staff cohorts fostered positive working relationships and promoted meaningful connection. It also allowed staff to better understand the needs of a particular group of residents and anticipate what specific support they could provide them during difficult times of the pandemic. These relationships encouraged positivity, teamwork, and helped reduce resident loneliness.

The findings suggest that staff appreciation was an important element in staff and resident well‐being during the pandemic. Multiple staff members reported that when they felt appreciated and supported by management, they felt more positive towards their work. This helped them better support and connect with residents, which helped mitigate resident loneliness. Managers appreciated staff by offering small gifts (e.g. individually wrapped baked goods with written thank‐you notes) and words of affirmation and encouragement. Managers also showed appreciation by verbally thanking each staff member for their hard work, checking‐in with staff members to see how they were doing, and ensuring managers were in‐person at the LTC home (instead of virtual) to prevent staff from feeling abandoned during the pandemic. Staff appreciation directly impacts resident well‐being because when staff felt supported and happy, it positively influenced the support residents received. Given the challenges the COVID‐19 pandemic imposes on LTC staff, they need to be uplifted and encouraged through difficult times so they can best support and ensure the well‐being of residents.

## 4 DISCUSSION

The first two themes emphasise the loneliness experienced among residents in LTC due to social isolation, missing family and friends, and grief. The second two themes describe how this loneliness was mitigated. By including both resident and staff perspectives, we were able to integrate critical perspectives to generate a better understanding of resident loneliness in LTC and summarise strategies of overcoming this loneliness.

### 4.1 Residents' experience of loneliness

This study suggests some of the reasons why the COVID‐19 pandemic exacerbated experiences of loneliness among residents in LTC. A significant factor was the visitor ban, which has been cited in other studies on this topic in Europe, Australia, the United States and other areas in Canada (Aho, 2022; Beogo et al., 2022; Rasnaca et al., 2022; Simard & Volicer, 2020; Van der Roest et al., 2020). This study provides qualitative data directly from residents and staff members to support that the visitor ban contributed to resident loneliness.

Findings in the study also shed light on the long‐term impact of loneliness and confinement on both the physical and mental health of residents. Confining residents to their room and limiting their contact with others can have devastating and potentially permanent health consequences, some that may be more extreme than if the resident was at risk for COVID‐19 infection. This is supported by work from Aho (2022), who argues ‘We're protecting [residents] to death’. Aho suggests the extreme measures taken to prevent spread of COVID‐19 has left residents ‘confused and abandoned to an existence that has been drained of meaning and significance’. This study supports Aho's perspective by providing stories from staff about residents ‘almost giving up’ and that they ‘didn't see the point’ regarding functioning and engagement in daily activities such as eating or leaving their room. Finding a balance between COVID‐19 public health measures and resident well‐being is essential moving forward in the pandemic (Van der Roest et al., 2020).

This study also brings attention to the impact of grief on resident loneliness. Most literature related to grief in LTC is focused on the grief of families losing their loved ones living in LTC (Cohen‐Mansfield & Meschiany, 2022; Tupper et al., 2020). Literature on grief experienced by residents in LTC and its impact on loneliness is lacking. Our findings provide evidence that resident's experiences of grief can have negative influences on their well‐being and needs to be considered when taking action to reduce loneliness in LTC during the COVID‐19 pandemic.

Notably, staff members perceived the levels and impact of loneliness to be more extreme on residents than what was described by some residents about their own experiences. This aligns with work from Van der Roest et al. (2020) who administered online surveys to residents and staff. Van der Roest et al. found 77% of residents revealed they experienced loneliness, while staff reported higher levels (81%) of resident loneliness. Staff also specifically reported two times more residents were ‘strongly lonely’ (31%) compared to resident's self‐reports of feeling ‘strongly lonely’ (16%) and that more residents were ‘very strongly lonely’ (16%) compared to resident's self‐reports of feeling ‘very strongly lonely’ (11%). This study provides some possible explanations as to why staff may perceive higher and more severe levels of resident loneliness. Residents may not feel comfortable expressing how lonely they are or try to underplay their experience, as resident Amanda suggested. Some residents may also have a different understanding of loneliness compared to staff. For example, resident Leo did not report feeling lonely because he had staff physically around him. This suggests he is equating loneliness to social isolation, which is a different concept (Silva et al., 2022). This is further supported by his desire to leave the care home to connect with friends in the community, suggesting the physical presence of people is not providing meaningful social connection.

### 4.2 Mitigating resident loneliness

The first two themes characterise the residents' experiences of loneliness. Examining the final two themes is an important step in understanding effective tools for mitigating loneliness in LTC during the pandemic. Theme 3 in particular highlights the effectiveness of providing different forms of social support depending on the resident's situation and social network. Social support may be in the form of socialising with staff, other residents, and/or family and friends remotely.

Social support should be encouraged as much as possible, particularly between residents and staff. When care homes were understaffed, staff became stressed and more task oriented, with little time to speak to residents (McCormack et al., 2010). Throughout the pandemic, it has been difficult for LTC homes across the world to maintain adequate staffing (Abbasi, 2020). Some of the biggest factors contributing to understaffing mentioned by the staff interviewed in this study included fear of exposure to COVID‐19, low pay and burnout from the extra workload. More temporary fluctuations in staffing also occurred due to a high proportion of frontline workers being exposed to COVID‐19 in the LTC home or testing positive and having to isolate at home for periods of time. One solution that healthcare authorities have implemented is creating a pool of staff who are on standby to replace LTC staff who are sick or were exposed to the virus. Standby staff may also be available to fill in empty positions until long‐term employees can be found. Continuing to pursue solutions to staff shortages is important because adequate staffing is important not only to ensure proper care, but also provide extra resources that can be dedicated to spending time with each resident to improve their quality of life.

Two staff members and a resident reported some staff members seem less confident or do not have the time to connect with residents. Although we do not know the reasoning behind why this may be the case for a subset of staff (e.g. do they struggle supporting residents when they are grieving in particular? Do they feel overwhelmed by their workload and are being task‐oriented? Are there language and/or cultural barriers between some staff and residents?), providing educational programs to learn about person‐centred care (Crandall et al., 2007) and tips on how to meaningfully connect with residents during their daily duties would likely be beneficial for all staff.

There may be times when staff do not have enough time to connect meaningfully with each resident. Thus, different methods of socialisation beyond care staff should be explored. For example, telepresence robots (Niemelä et al., 2021) and social robots (Hung et al., 2019) have been reported as beneficial to promote connectedness in LTC. Virtual socialisation should continue to be supported in LTC as it has shown to be successful in connecting older adults with friends and family during the COVID‐19 pandemic (Brooke et al., 2022). It can be implemented during both outbreak and non‐outbreak settings, and even after the pandemic to connect residents with loved ones who live far away. Furthermore, matching residents to volunteers or paid companions who can connect with them virtually is another tool to maintain connection during outbreak and non‐outbreak settings.

Levels of social connection were also impacted by the physical environment. For example, having windows in communal areas and resident's rooms facilitated window visits to allow safe social connection during outbreak and non‐outbreak settings. Furthermore, the small home structure allowed ‘bubbles’ to form within neighbourhoods to permit modified forms of recreation and socialisation when there was no outbreak in the neighbourhood. The layout of the building also impacted the accessibility of Wi‐Fi, which affected the ability of residents to participate in video calls throughout the pandemic.

The particular neighbourhood residents lived in also impacted their level of socialisation. Placing residents on a floor with people who speak the same language and have similar cognitive abilities may help promote socialisation when social activity is not restricted due to an outbreak. An alternative is establishing peer‐to‐peer matching among residents based on similar interests and functioning.

Furthermore, finding a safe way to permit visits throughout the pandemic is important in connecting residents with their loved ones. It is difficult to allow visits during outbreaks (with the exception of window visits that can be done safely); however, whenever possible, LTC homes should promote visitation during non‐outbreak settings. For example, developing a pressurised room may help facilitate visitation while reducing the risk of infection (Al‐Benna, 2021). Outdoor visits may also be facilitated by placing chairs a safe distance apart outside. Finally, rapid COVID‐19 testing is also an option to allow visitors with negative tests to enter the care home.

Theme 4 highlighted the effectiveness of creating opportunities for recreation and fostering a positive atmosphere in reducing resident loneliness. Modified recreational activities provided events and activities that residents could look forward to and enjoy. The activities listed in Table 3, and their respective settings (outbreak vs. non‐outbreak settings) should be considered for implementation during COVID‐19. Investigating other tools that provide safe and meaningful leisure during outbreak and non‐outbreak settings, such as Ambient Activity Technology (Wilkinson et al., 2017), may be beneficial. Ambient Activity Technology is a device that can be kept in a resident's room and is designed to engage residents with self‐accessed and personalised interactions at any time. For example, resident can use the touch screen and listen to the audio instructions that guide interactions (e.g. ‘match the pictures’).

The sources of a positive atmosphere included positive morale among both residents and staff, a positive physical atmosphere (‘They should feel that it's warm and welcoming than just a hospital’), inclusivity, staff cohorts and staff appreciation. Since residents emphasised the importance of staying positive to avoid feeling lonely, finding ways to boost resident morale may be helpful in both outbreak and non‐outbreak settings. For example, care homes could encourage members of the public to send in handwritten cards, letters and videos for residents (Brown, 2020) focused on encouragement. Offering residents decorations (e.g. art or positive messages) for their rooms could help boost positivity and also contribute to a more positive physical environment, especially in situations where residents are confined to their room.

Encouraging staff to work in small and consistent cohorts may help promote a positive atmosphere for both staff and residents as they can get to know each other and develop closer relationships, particularly during outbreak settings. In LTC homes that have the small home model (i.e. smaller groups of buildings rather than one large shared building), staff cohorts may be assembled for each building. For LTC homes that have one large shared building, staff may be assembled into small groups that work consistently on the same floor or section of the building. This was suggested to better promote relationship building and mitigate COVID‐19 spread compared to changing the location in the building where staff members work each shift.

In addition, staff should be appreciated by management and other stakeholders in LTC, particularly when they dedicate time to know the residents and improve residents' quality of life. To evaluate whether staff feel appreciated and supported, they should be asked directly. For example, staff may fill out an anonymous feedback form that assesses (1) if they feel supported by management, and (2) what are some of the actions management could take to make them feel more supported. Management should later meet to discuss the feedback and plan for improvements or continuation of positive actions. Staff and management should also be encouraged to focus on inclusivity, such as appreciating different cultures across residents and avoiding activities that make certain residents feel excluded. For example, when celebrating holidays, staff should ensure all cultures of residents are being reflected. One of the largest holiday celebrations in North America is Christmas; however, residents who are Jewish or Buddhist may feel not feel represented without a celebration of Hanukkah and Bodhi Day, which also fall in December. Staff should survey their resident population, and perhaps ask residents themselves which cultural celebrations they would like to celebrate and adapt the holiday calendar to improve inclusivity.

Since grief counselling benefited residents, implementing formal mental health support (e.g. with a virtual counsellor or spiritual care worker) may help mitigate loneliness and promote well‐being throughout the pandemic. This is also important because individuals with serious mental illness are overrepresented in LTC and expected to increase in coming years even though many frontline staff lack the training to support this population (Muralidharan et al., 2019).

These themes expand on Simard and Volicer's (2020) work, who provided a list of recommendations to reduce resident loneliness in LTC. They recommended ideas to mitigate loneliness that have some similarities to this work, such as matching volunteers with residents remotely to facilitate social connection or encouraging modified recreation (e.g. connecting with religious service virtually). However, Simard and Volicer (2020) did not interview residents or staff directly to understand resident loneliness. Instead, they summarised the existing literature related to the impact of COVID‐19 on loneliness and isolation in LTC to offer solutions to improve the quality of life of residents. It is unclear if any of the suggestions were informed by staff or residents, or if any were successful in practice. Although the suggestions from Simard and Volicer may be useful, the findings in this work reveal what tools were successful in practice to mitigate loneliness and demonstrate what initiatives reflect what residents value and believe are important. The suggestions provided in Themes 3 and 4 should be considered alongside Simard and Volicer's (2020) work when selecting and implementing new initiatives in LTC homes to mitigate resident loneliness.

Recent literature also focuses on methods to connect residents with loved ones during the COVID‐19 pandemic (Forma et al., 2020; Wong et al., 2020). Ickert et al. (2020) report technology is one of the most commonly promoted methods for maintaining social connections during COVID‐19 in Alberta, Canada. They emphasise the importance of accessible Wi‐Fi throughout the care home and the need for staff assistance to set up calls for some residents. This aligns with our findings and highlights the importance of not only making tablets available for residents to use, but also ensuring they can connect to Wi‐Fi from their rooms and dedicating resources (staff or volunteers) to help set up calls for residents. Monin et al. (2020) presented data from a nationally targeted online survey and reported familiar methods such as phone calls and emails between families and LTC residents helped maintain well‐being during periods of visitation ban in the United States. Some residents in this study also preferred using a telephone over a tablet, suggesting older methods of connection may be more successful for certain residents.

Although interviews were conducted in context of the COVID‐19 pandemic, many of the above recommendations on mitigating residents' loneliness may be modified and applied to non‐pandemic settings. For example, mental health support may be offered in‐person instead of virtual in a post‐pandemic setting. Since resident loneliness was present prior to the COVID‐19 pandemic, implementing changes in LTC to mitigate loneliness beyond the pandemic may benefit residents in the long‐term.

### 4.3 Limitations

A limitation of this study is a potential lack of transferability. Since participants were only recruited from two LTC sites in the Vancouver area, the results may not reflect the experiences of all residents in LTC. Interviews were conducted only in English, so perspectives from potential participants who were not comfortable speaking English were not captured. These residents may have had different experiences with loneliness, particularly because they may not share a language with staff members and feel further isolated. Furthermore, including more participants, as well as participants (both staff and residents) with a more diverse set of demographics (e.g. racial and ethnic backgrounds, socioeconomic status, sex, gender and age), may have allowed the collection of a larger range of experiences. Finally, holding interviews closer to the major outbreaks may have permitted more detailed stories of the experience, although efforts were made to conduct the interviews as early as possible.

### 4.4 Contribution to the literature

Our study shows the important perspectives of residents and is supplemented by perspectives of a large range of staff in LTC (nurses, care workers, music therapists, recreation staff, administrators and directors). As shown in the results, residents and staff perspectives differ and information can be missed if a research team only focuses on one group in LTC. In addition, half of our work was dedicated to characterising the loneliness experienced by residents, while the other half focused on ways this loneliness can be mitigated. This is an important piece of the work because we asked residents and staff directly what was effective in mitigating loneliness and proposed many tools that align with these perspectives.

Our study is also the first study in North America to our knowledge that interviewed staff and residents directly to understand experiences of resident loneliness and report what helped mitigate it during the COVID‐19 pandemic. The COVID‐19 impact and isolation protocols across regions differ, as well as the socioeconomic and cultural factors that impact residents' experiences of loneliness. Although there were similarities in findings with other studies, the residents and staff included in this study also had differing perspectives, experiences and ideas compared to studies conducted in other regions. These differences may relate to country of origin and the experiences and setting accompanied with it. Thus, the Canadian perspective and context on this topic is important to contribute to the literature.

Our study provides a holistic view of the effective mechanisms for overcoming loneliness to inform staff, management, other researchers and the public. However, the issue of social isolation and loneliness in LTC existed prior to the pandemic (Andrew & Meeks, 2018) Thus, this work is also relevant to a post‐pandemic setting. The stories and experiences described in this work should be closely examined, particularly when creating new infection control protocols, support programs and educational programs for staff.

Given that the COVID‐19 pandemic is related to climate change, environmental policy makers can play a role to improve the well‐being of residents in LTC. Many root causes of climate change, such as deforestation, can increase the risk of pandemics (Tollefson, 2020). Climate change can also exacerbate the impact of an infectious disease. In the context of COVID‐19, a recent study found an association between long‐term exposure to air pollution and higher COVID‐19 mortality rates (Wu et al., 2020). Thus, climate change can increase the risk of spread and mortality of infectious diseases. This results in a need for public health measures (e.g. social isolation and social distancing) and increased likelihood of survivors grieving for a loved one who died from the disease, two factors that were found in this study to increase levels of loneliness among residents in LTC. Policy makers should focus on preventing future pandemics and reducing the impact of the current COVID‐19 pandemic. To do this, policy makers may prioritise efforts to mitigate greenhouse gas emissions and other contributors to climate change. Slowing down or reversing climate change may decrease the likelihood of future pandemics and consequently mitigate the risk of increased loneliness experienced by LTC residents.

Future research should focus on investigating and testing different tools to mitigate loneliness and include larger sample sizes from a diverse range of LTC sites. The results of this study will be considered in the next phase of the CAR process: working with stakeholders to create and implement new policy, educational tools, and changes to practice in LTC homes.

## 5 CONCLUSION

This qualitative study highlights the feelings of social isolation, missing family and friends, and grief that contributed to the loneliness experienced by older adults living in LTC during the pandemic. Residents continue to be at risk of loneliness unless interventions are put in place such as promoting social support, opportunities for recreation and positivity in LTC homes. Resident loneliness will not be resolved using one tool, but may be mitigated by a combination of different supports and services involving staff, management, volunteers and technology—all with the goal of enhancing the quality of life among residents during the pandemic.

## 6 IMPLICATIONS FOR PRACTICE

- Highlights the impact of social isolation, missing family and friends, and grief on resident loneliness to better understand residents' experiences of loneliness in LTC throughout the COVID‐19 pandemic
- Emphasises the importance of providing different forms of social support, creating opportunities for recreation and promoting a positive atmosphere in LTC settings to help mitigate resident loneliness.
- Influences policy and practice by providing a summary of resident and staff experiences to inform decisions related to new infection control protocols, support programs, and educational programs for staff.
- Encourages stakeholders in LTC to implement new incentives that provide social support, recreation opportunities and positivity to mitigate resident loneliness.
- Provides a foundation for future research focusing on investigating and testing different tools to mitigate resident loneliness in LTC.

## CONFLICT OF INTEREST

There are no conflicts of interest.

## Supporting information


Appendix A1

## Data Availability

The data that support the findings of this study are available from the corresponding author upon reasonable request.
