# Supplementary material for: Exploring experiences of loneliness among Canadian long‐term care residents during the COVID‐19 pandemic: A qualitative study
Source: Int J Older People Nurs. 2022 Nov 8;18(1):e12509. doi: 10.1111/opn.12509 (PMC9878008; doi:10.1111/opn.12509)
Supplement: Supplementary file 1 — Appendix A1 [file OPN-18-0-s001.docx]

Appendix A

1. Interview questions.

For the residents, we asked: (1) “What was it like for you during the COVID-19 outbreak?” (2) “Tell me a story about a time when you felt lonely during the pandemic.” (3) “What helped you to alleviate the feeling of loneliness?” (4) “Did the staff do anything to help you feel less lonely?” (5) “Do you connect with any family or friends outside of the home?” (6) “What can be done to reduce loneliness in your care home?”

For the staff, we asked: (1) “Can you tell me a story about your care experience during the pandemic related to loneliness of residents?” (2) “What were some things you did to reduce loneliness for the residents? What are the challenges?” (3) “What resources do you need to mitigate loneliness among residents?”

1. Quotation from staff member about resident loneliness.

‘There is this one resident she is very able, ambulatory, no mobility aids. She is well and healthy but when the lockdown started well, she's used to having family over to visit her especially her daughter ... So during the lockdown, they weren't able to do that so what happened was the resident started not to eat. She started not going out of her room at all, lying down on the bed almost all the time … eventually because of not eating well just being lonely and looking for her daughter eventually she declined and unfortunately, she also passed away during the lockdown. And then another one is another resident also very able and friendly gentleman, always looking for his daughter and wanting to go home. But then because of lockdown they're not able to see each other and he felt that a lot and eventually declined and also died.’
